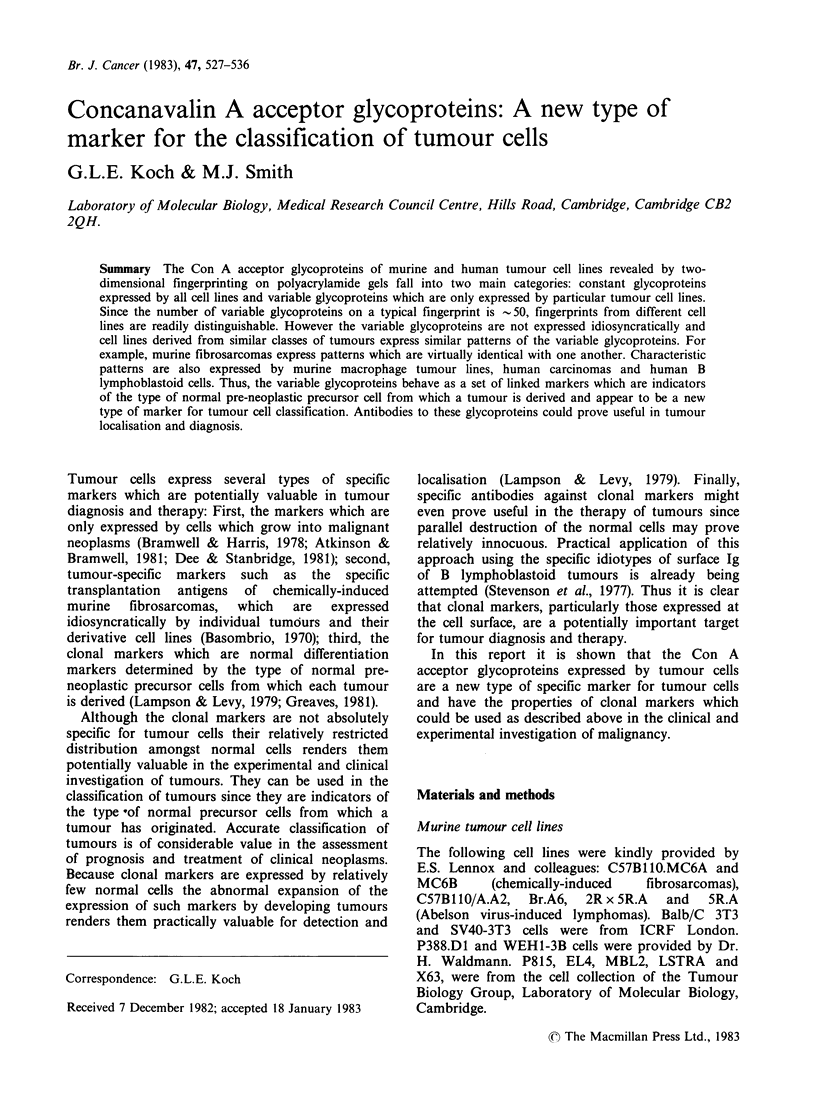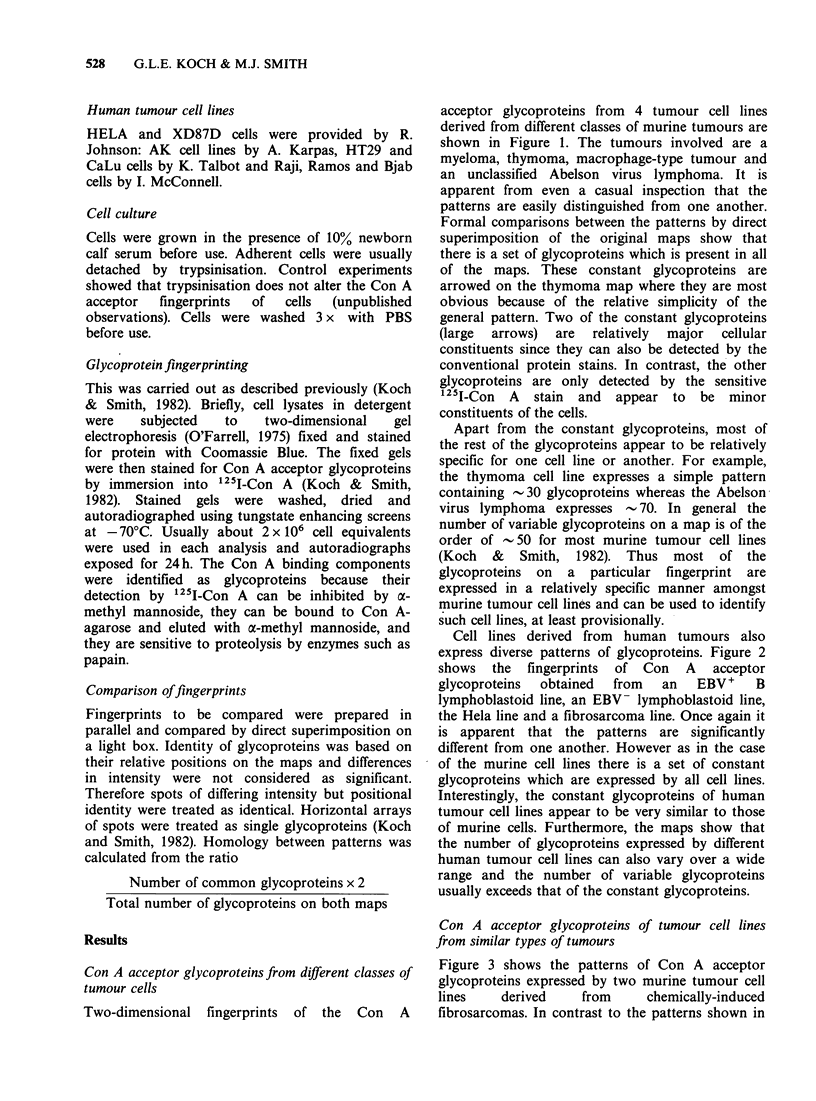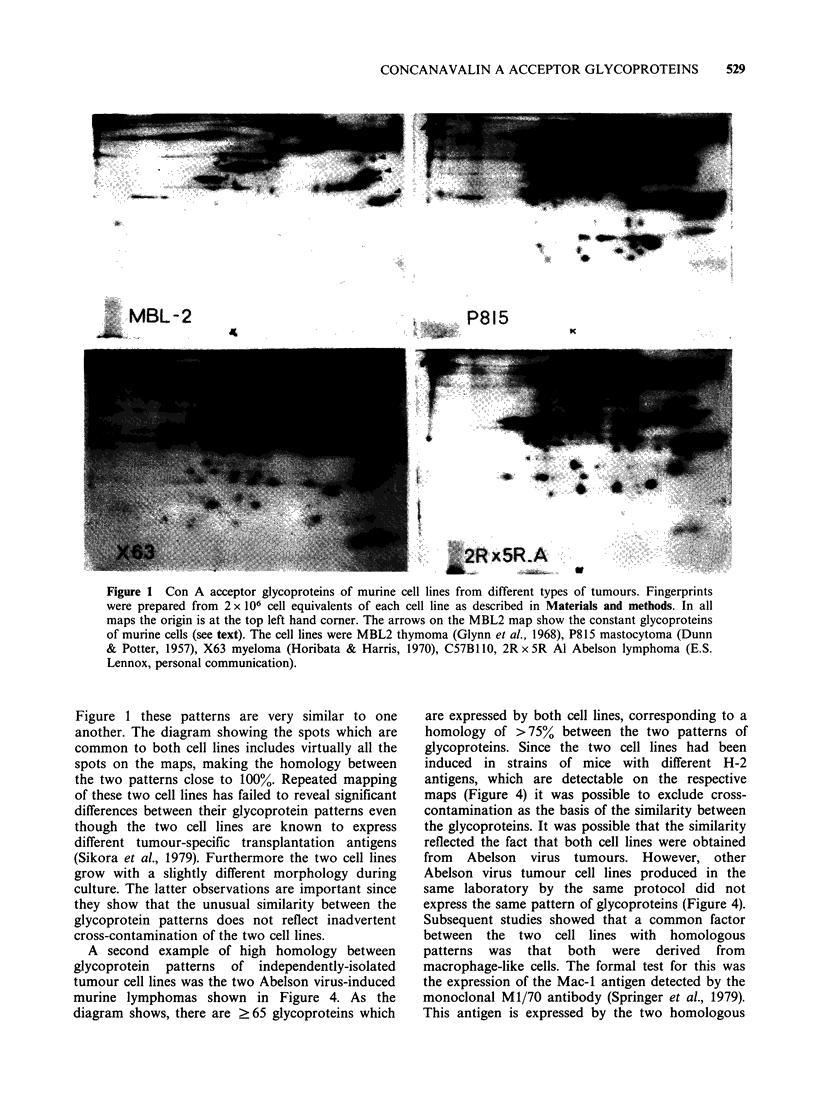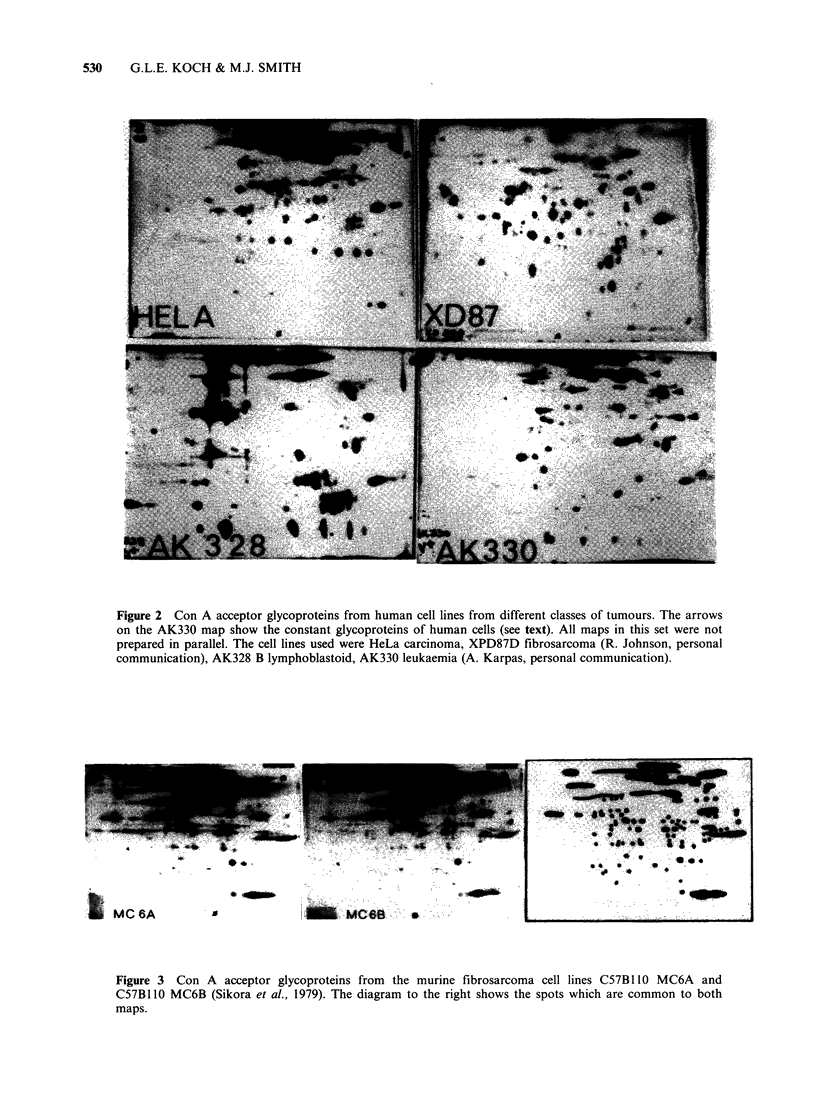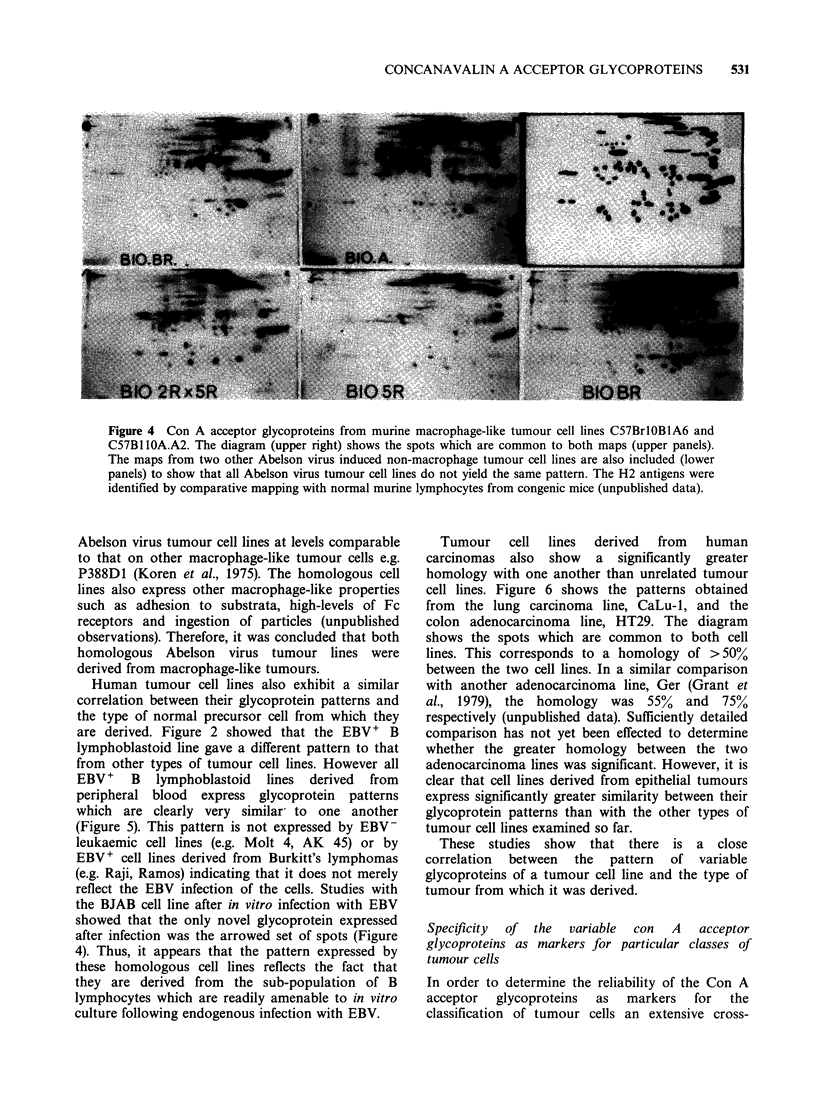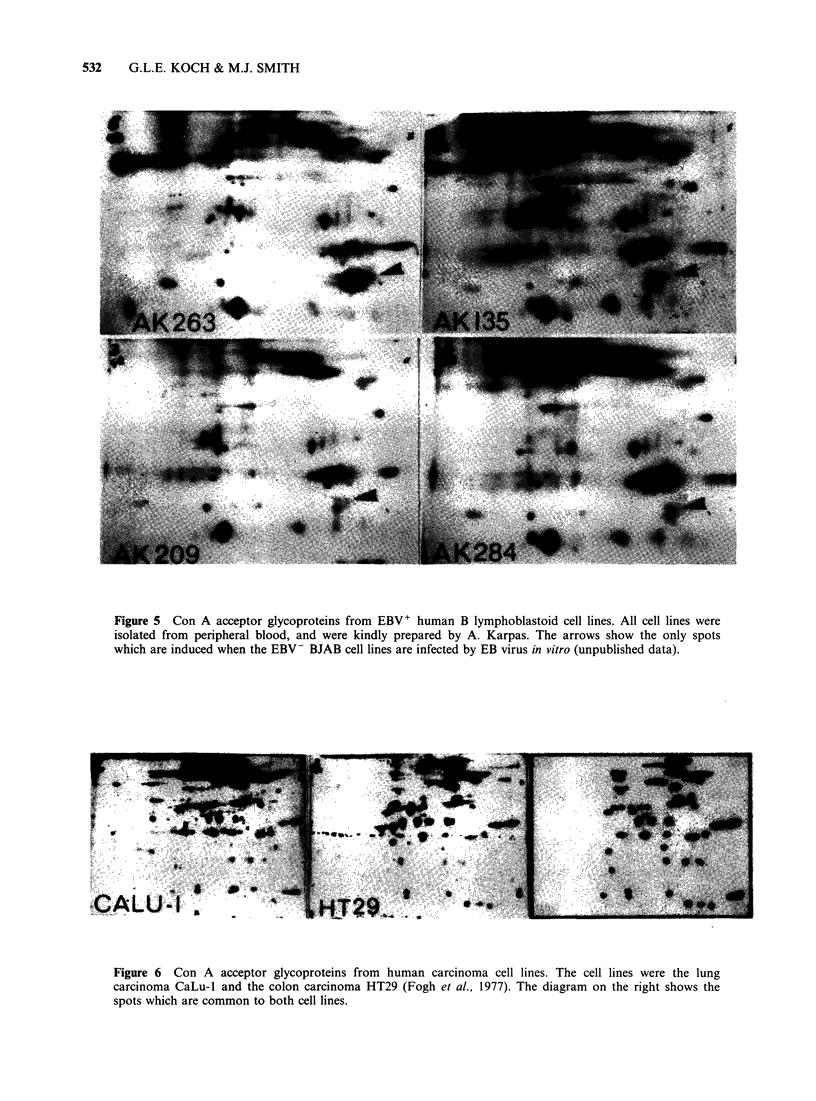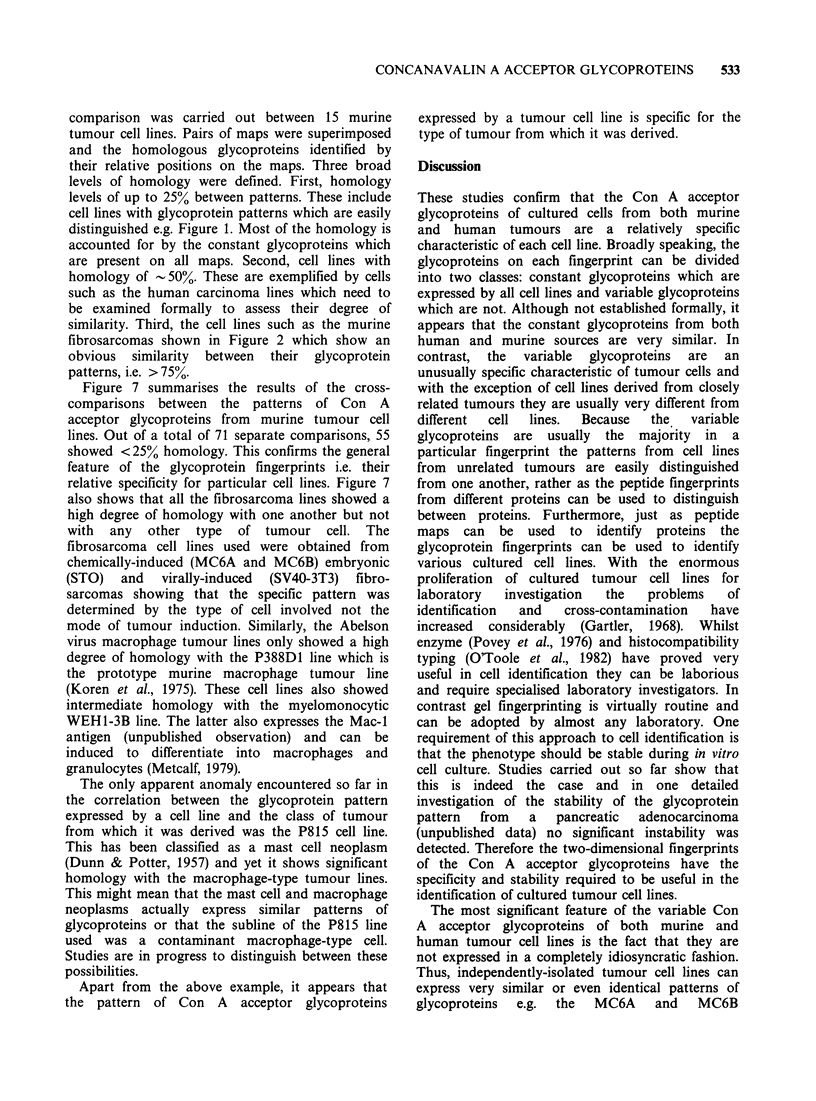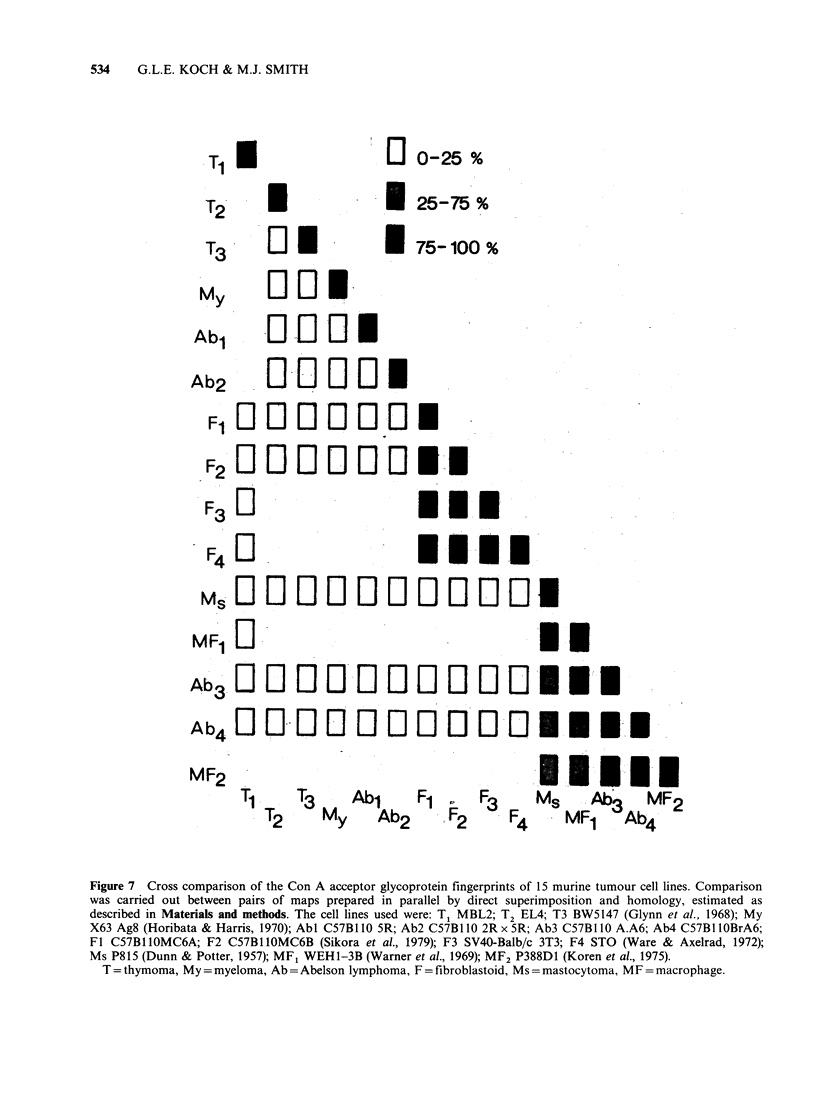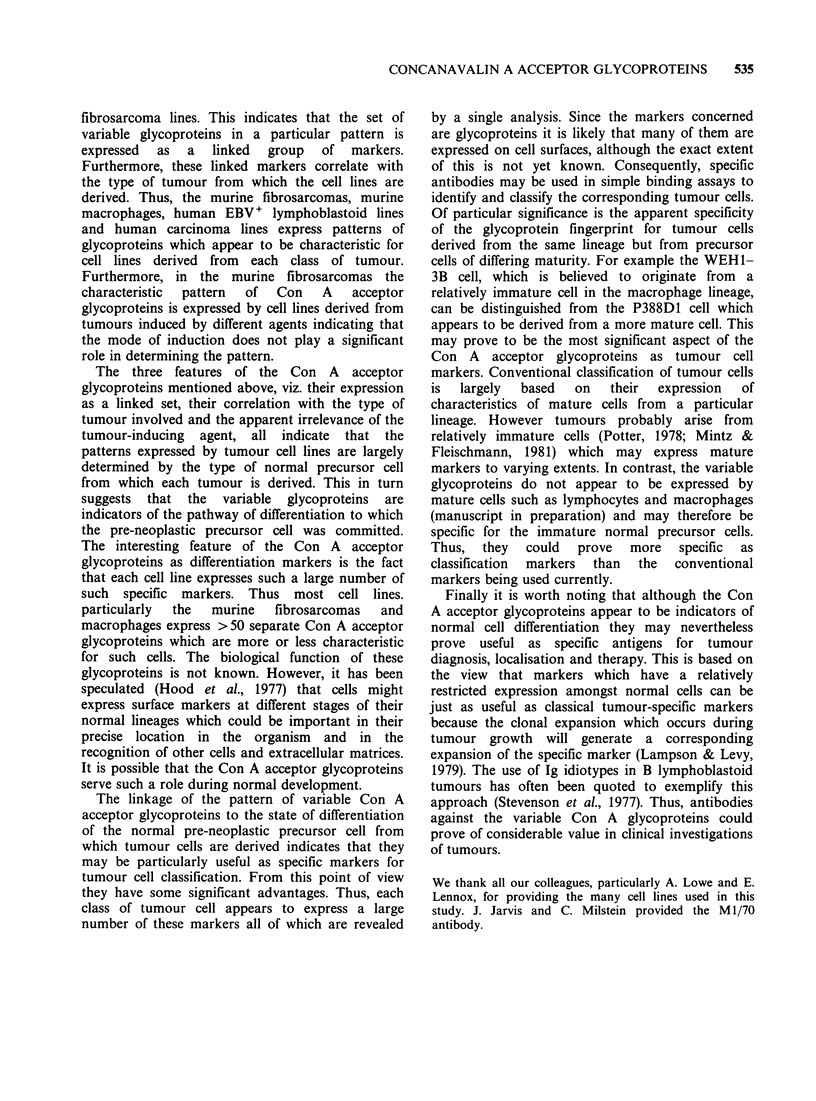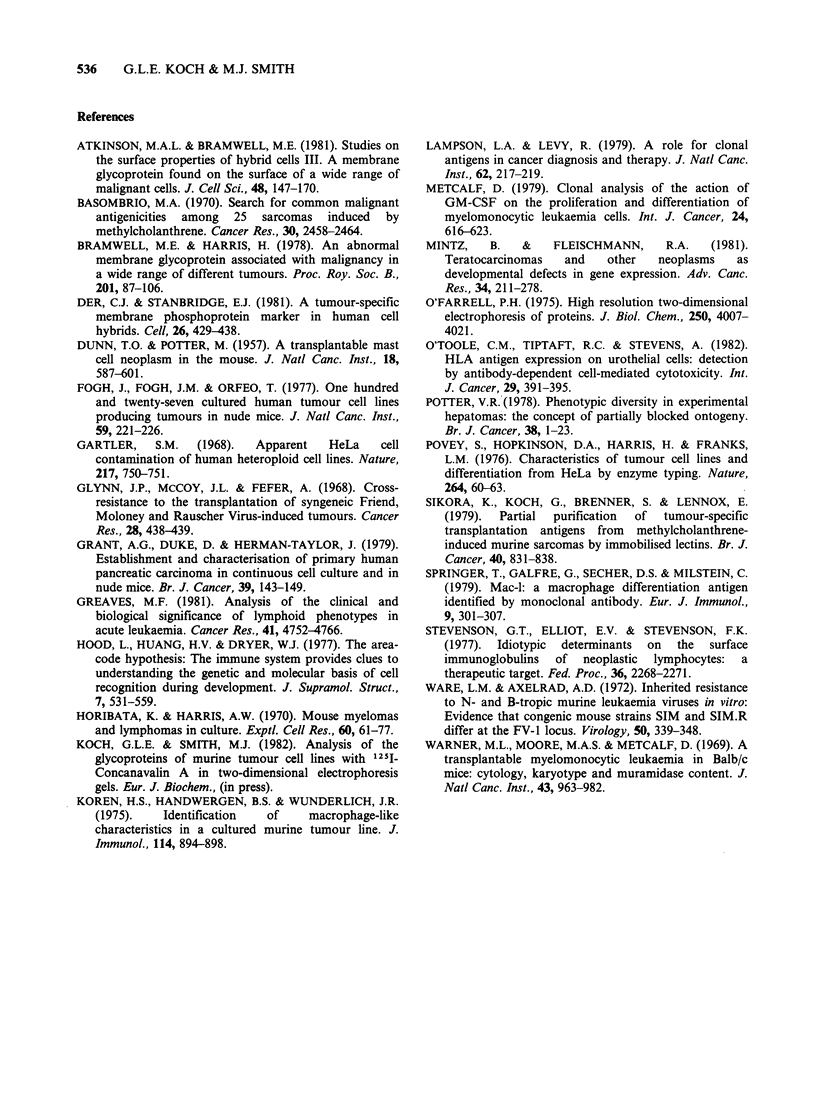# Concanavalin A acceptor glycoproteins: a new type of marker for the classification of tumour cells.

**DOI:** 10.1038/bjc.1983.83

**Published:** 1983-04

**Authors:** G. L. Koch, M. J. Smith

## Abstract

**Images:**


*Br. J. Cancer* (1983), **47**, 527–536

### Concanavalin A acceptor glycoproteins: A new type of marker for the classification of tumour cells

G.L.E. Koch & M.J. Smith

*Laboratory of Molecular Biology, Medical Research Council Centre, Hills Road, Cambridge, Cambridge CB2 2QH.*

**Summary** The Con A acceptor glycoproteins of murine and human tumour cell lines revealed by two-dimensional fingerprinting on polyacrylamide gels fall into two main categories: constant glycoproteins expressed by all cell lines and variable glycoproteins which are only expressed by particular tumour cell lines. Since the number of variable glycoproteins on a typical fingerprint is ~50, fingerprints from different cell lines are readily distinguishable. However the variable glycoproteins are not expressed idiosyncratically and cell lines derived from similar classes of tumours express similar patterns of the variable glycoproteins. For example, murine fibrosarcomas express patterns which are virtually identical with one another. Characteristic patterns are also expressed by murine macrophage tumour lines, human carcinomas and human B lymphoblastoid cells. Thus, the variable glycoproteins behave as a set of linked markers which are indicators of the type of normal pre-neoplastic precursor cell from which a tumour is derived and appear to be a new type of marker for tumour cell classification. Antibodies to these glycoproteins could prove useful in tumour localisation and diagnosis.

Tumour cells express several types of specific markers which are potentially valuable in tumour diagnosis and therapy: First, the markers which are only expressed by cells which grow into malignant neoplasms (Bramwell & Harris, 1978; Atkinson & Bramwell, 1981; Dee & Stanbridge, 1981); second, tumour-specific markers such as the specific transplantation antigens of chemically-induced murine fibrosarcomas, which are expressed idiosyncratically by individual tumours and their derivative cell lines (Basombrio, 1970); third, the clonal markers which are normal differentiation markers determined by the type of normal pre-neoplastic precursor cells from which each tumour is derived (Lampson & Levy, 1979; Greaves, 1981).

Although the clonal markers are not absolutely specific for tumour cells their relatively restricted distribution amongst normal cells renders them potentially valuable in the experimental and clinical investigation of tumours. They can be used in the classification of tumours since they are indicators of the type of normal precursor cells from which a tumour has originated. Accurate classification of tumours is of considerable value in the assessment of prognosis and treatment of clinical neoplasms. Because clonal markers are expressed by relatively few normal cells the abnormal expansion of the expression of such markers by developing tumours renders them practically valuable for detection and localisation (Lampson & Levy, 1979). Finally, specific antibodies against clonal markers might even prove useful in the therapy of tumours since parallel destruction of the normal cells may prove relatively innocuous. Practical application of this approach using the specific idiotypes of surface Ig of B lymphoblastoid tumours is already being attempted (Stevenson *et al.*, 1977). Thus it is clear that clonal markers, particularly those expressed at the cell surface, are a potentially important target for tumour diagnosis and therapy.

In this report it is shown that the Con A acceptor glycoproteins expressed by tumour cells are a new type of specific marker for tumour cells and have the properties of clonal markers which could be used as described above in the clinical and experimental investigation of malignancy.

### Materials and methods

### *Murine tumour cell lines*

The following cell lines were kindly provided by E.S. Lennox and colleagues: C57B110.MC6A and MC6B (chemically-induced fibrosarcomas), C57B110/A.A2, Br.A6, 2R × 5R.A and 5R.A (Abelson virus-induced lymphomas). Balb/C 3T3 and SV40-3T3 cells were from ICRF London. P388.D1 and WEH1-3B cells were provided by Dr. H. Waldmann. P815, EL4, MBL2, LSTRA and X63, were from the cell collection of the Tumour Biology Group, Laboratory of Molecular Biology, Cambridge.

Correspondence: G.L.E. Koch

Received 7 December 1982; accepted 18 January 1983

© The Macmillan Press Ltd., 1983

*Human tumour cell lines*

HELA and XD87D cells were provided by R. Johnson: AK cell lines by A. Karpas, HT29 and CaLu cells by K. Talbot and Raji, Ramos and Bjab cells by I. McConnell.

*Cell culture*

Cells were grown in the presence of 10% newborn calf serum before use. Adherent cells were usually detached by trypsinisation. Control experiments showed that trypsinisation does not alter the Con A acceptor fingerprints of cells (unpublished observations). Cells were washed 3× with PBS before use.

*Glycoprotein fingerprinting*

This was carried out as described previously (Koch & Smith, 1982). Briefly, cell lysates in detergent were subjected to two-dimensional gel electrophoresis (O'Farrell, 1975) fixed and stained for protein with Coomassie Blue. The fixed gels were then stained for Con A acceptor glycoproteins by immersion into $^{125}$I-Con A (Koch & Smith, 1982). Stained gels were washed, dried and autoradiographed using tungstate enhancing screens at −70°C. Usually about $2\times10^6$ cell equivalents were used in each analysis and autoradiographs exposed for 24 h. The Con A binding components were identified as glycoproteins because their detection by $^{125}$I-Con A can be inhibited by α-methyl mannoside, they can be bound to Con A-agarose and eluted with α-methyl mannoside, and they are sensitive to proteolysis by enzymes such as papain.

*Comparison of fingerprints*

Fingerprints to be compared were prepared in parallel and compared by direct superimposition on a light box. Identity of glycoproteins was based on their relative positions on the maps and differences in intensity were not considered as significant. Therefore spots of differing intensity but positional identity were treated as identical. Horizontal arrays of spots were treated as single glycoproteins (Koch and Smith, 1982). Homology between patterns was calculated from the ratio

$$\frac{\text{Number of common glycoproteins} \times 2}{\text{Total number of glycoproteins on both maps}}$$

### Results

*Con A acceptor glycoproteins from different classes of tumour cells*

Two-dimensional fingerprints of the Con A acceptor glycoproteins from 4 tumour cell lines derived from different classes of murine tumours are shown in Figure 1. The tumours involved are a myeloma, thymoma, macrophage-type tumour and an unclassified Abelson virus lymphoma. It is apparent from even a casual inspection that the patterns are easily distinguished from one another. Formal comparisons between the patterns by direct superimposition of the original maps show that there is a set of glycoproteins which is present in all of the maps. These constant glycoproteins are arrowed on the thymoma map where they are most obvious because of the relative simplicity of the general pattern. Two of the constant glycoproteins (large arrows) are relatively major cellular constituents since they can also be detected by the conventional protein stains. In contrast, the other glycoproteins are only detected by the sensitive $^{125}$I-Con A stain and appear to be minor constituents of the cells.

Apart from the constant glycoproteins, most of the rest of the glycoproteins appear to be relatively specific for one cell line or another. For example, the thymoma cell line expresses a simple pattern containing ∼30 glycoproteins whereas the Abelson virus lymphoma expresses ∼70. In general the number of variable glycoproteins on a map is of the order of ∼50 for most murine tumour cell lines (Koch & Smith, 1982). Thus most of the glycoproteins on a particular fingerprint are expressed in a relatively specific manner amongst murine tumour cell lines and can be used to identify such cell lines, at least provisionally.

Cell lines derived from human tumours also express diverse patterns of glycoproteins. Figure 2 shows the fingerprints of Con A acceptor glycoproteins obtained from an EBV$^+$ B lymphoblastoid line, an EBV$^-$ lymphoblastoid line, the Hela line and a fibrosarcoma line. Once again it is apparent that the patterns are significantly different from one another. However as in the case of the murine cell lines there is a set of constant glycoproteins which are expressed by all cell lines. Interestingly, the constant glycoproteins of human tumour cell lines appear to be very similar to those of murine cells. Furthermore, the maps show that the number of glycoproteins expressed by different human tumour cell lines can also vary over a wide range and the number of variable glycoproteins usually exceeds that of the constant glycoproteins.

*Con A acceptor glycoproteins of tumour cell lines from similar types of tumours*

Figure 3 shows the patterns of Con A acceptor glycoproteins expressed by two murine tumour cell lines derived from chemically-induced fibrosarcomas. In contrast to the patterns shown in

**Figure 1** Con A acceptor glycoproteins of murine cell lines from different types of tumours. Fingerprints were prepared from $2 \times 10^6$ cell equivalents of each cell line as described in **Materials and methods**. In all maps the origin is at the top left hand corner. The arrows on the MBL2 map show the constant glycoproteins of murine cells (see **text**). The cell lines were MBL2 thymoma (Glynn *et al.*, 1968), P815 mastocytoma (Dunn & Potter, 1957), X63 myeloma (Horibata & Harris, 1970), C57B110, 2R x 5R Al Abelson lymphoma (E.S. Lennox, personal communication).

Figure 1 these patterns are very similar to one another. The diagram showing the spots which are common to both cell lines includes virtually all the spots on the maps, making the homology between the two patterns close to 100%. Repeated mapping of these two cell lines has failed to reveal significant differences between their glycoprotein patterns even though the two cell lines are known to express different tumour-specific transplantation antigens (Sikora *et al.*, 1979). Furthermore the two cell lines grow with a slightly different morphology during culture. The latter observations are important since they show that the unusual similarity between the glycoprotein patterns does not reflect inadvertent cross-contamination of the two cell lines.

A second example of high homology between glycoprotein patterns of independently-isolated tumour cell lines was the two Abelson virus-induced murine lymphomas shown in Figure 4. As the diagram shows, there are $\geq 65$ glycoproteins which are expressed by both cell lines, corresponding to a homology of >75% between the two patterns of glycoproteins. Since the two cell lines had been induced in strains of mice with different H-2 antigens, which are detectable on the respective maps (Figure 4) it was possible to exclude cross-contamination as the basis of the similarity between the glycoproteins. It was possible that the similarity reflected the fact that both cell lines were obtained from Abelson virus tumours. However, other Abelson virus tumour cell lines produced in the same laboratory by the same protocol did not express the same pattern of glycoproteins (Figure 4). Subsequent studies showed that a common factor between the two cell lines with homologous patterns was that both were derived from macrophage-like cells. The formal test for this was the expression of the Mac-1 antigen detected by the monoclonal M1/70 antibody (Springer *et al.*, 1979). This antigen is expressed by the two homologous

**Figure 2** Con A acceptor glycoproteins from human cell lines from different classes of tumours. The arrows on the AK330 map show the constant glycoproteins of human cells (see text). All maps in this set were not prepared in parallel. The cell lines used were HeLa carcinoma, XPD87D fibrosarcoma (R. Johnson, personal communication), AK328 B lymphoblastoid, AK330 leukaemia (A. Karpas, personal communication).

**Figure 3** Con A acceptor glycoproteins from the murine fibrosarcoma cell lines C57B110 MC6A and C57B110 MC6B (Sikora *et al.*, 1979). The diagram to the right shows the spots which are common to both maps.

**Figure 4** Con A acceptor glycoproteins from murine macrophage-like tumour cell lines C57Br10B1A6 and C57B110A.A2. The diagram (upper right) shows the spots which are common to both maps (upper panels). The maps from two other Abelson virus induced non-macrophage tumour cell lines are also included (lower panels) to show that all Abelson virus tumour cell lines do not yield the same pattern. The H2 antigens were identified by comparative mapping with normal murine lymphocytes from congenic mice (unpublished data).

Abelson virus tumour cell lines at levels comparable to that on other macrophage-like tumour cells e.g. P388D1 (Koren *et al.*, 1975). The homologous cell lines also express other macrophage-like properties such as adhesion to substrata, high-levels of Fc receptors and ingestion of particles (unpublished observations). Therefore, it was concluded that both homologous Abelson virus tumour lines were derived from macrophage-like tumours.

Human tumour cell lines also exhibit a similar correlation between their glycoprotein patterns and the type of normal precursor cell from which they are derived. Figure 2 showed that the $EBV^+$ B lymphoblastoid line gave a different pattern to that from other types of tumour cell lines. However all $EBV^+$ B lymphoblastoid lines derived from peripheral blood express glycoprotein patterns which are clearly very similar to one another (Figure 5). This pattern is not expressed by $EBV^-$ leukaemic cell lines (e.g. Molt 4, AK 45) or by $EBV^+$ cell lines derived from Burkitt's lymphomas (e.g. Raji, Ramos) indicating that it does not merely reflect the EBV infection of the cells. Studies with the BJAB cell line after *in vitro* infection with EBV showed that the only novel glycoprotein expressed after infection was the arrowed set of spots (Figure 4). Thus, it appears that the pattern expressed by these homologous cell lines reflects the fact that they are derived from the sub-population of B lymphocytes which are readily amenable to *in vitro* culture following endogenous infection with EBV.

Tumour cell lines derived from human carcinomas also show a significantly greater homology with one another than unrelated tumour cell lines. Figure 6 shows the patterns obtained from the lung carcinoma line, CaLu-1, and the colon adenocarcinoma line, HT29. The diagram shows the spots which are common to both cell lines. This corresponds to a homology of $>50\%$ between the two cell lines. In a similar comparison with another adenocarcinoma line, Ger (Grant *et al.*, 1979), the homology was 55% and 75% respectively (unpublished data). Sufficiently detailed comparison has not yet been effected to determine whether the greater homology between the two adenocarcinoma lines was significant. However, it is clear that cell lines derived from epithelial tumours express significantly greater similarity between their glycoprotein patterns than with the other types of tumour cell lines examined so far.

These studies show that there is a close correlation between the pattern of variable glycoproteins of a tumour cell line and the type of tumour from which it was derived.

### *Specificity of the variable con A acceptor glycoproteins as markers for particular classes of tumour cells*

In order to determine the reliability of the Con A acceptor glycoproteins as markers for the classification of tumour cells an extensive cross-

**Figure 5** Con A acceptor glycoproteins from $EBV^+$ human B lymphoblastoid cell lines. All cell lines were isolated from peripheral blood, and were kindly prepared by A. Karpas. The arrows show the only spots which are induced when the $EBV^-$ BJAB cell lines are infected by EB virus *in vitro* (unpublished data).

**Figure 6** Con A acceptor glycoproteins from human carcinoma cell lines. The cell lines were the lung carcinoma CaLu-1 and the colon carcinoma HT29 (Fogh *et al.*, 1977). The diagram on the right shows the spots which are common to both cell lines.

comparison was carried out between 15 murine tumour cell lines. Pairs of maps were superimposed and the homologous glycoproteins identified by their relative positions on the maps. Three broad levels of homology were defined. First, homology levels of up to 25% between patterns. These include cell lines with glycoprotein patterns which are easily distinguished e.g. Figure 1. Most of the homology is accounted for by the constant glycoproteins which are present on all maps. Second, cell lines with homology of $\sim 50\%$. These are exemplified by cells such as the human carcinoma lines which need to be examined formally to assess their degree of similarity. Third, the cell lines such as the murine fibrosarcomas shown in Figure 2 which show an obvious similarity between their glycoprotein patterns, i.e. $>75\%$.

Figure 7 summarises the results of the cross-comparisons between the patterns of Con A acceptor glycoproteins from murine tumour cell lines. Out of a total of 71 separate comparisons, 55 showed $<25\%$ homology. This confirms the general feature of the glycoprotein fingerprints i.e. their relative specificity for particular cell lines. Figure 7 also shows that all the fibrosarcoma lines showed a high degree of homology with one another but not with any other type of tumour cell. The fibrosarcoma cell lines used were obtained from chemically-induced (MC6A and MC6B) embryonic (STO) and virally-induced (SV40-3T3) fibrosarcomas showing that the specific pattern was determined by the type of cell involved not the mode of tumour induction. Similarly, the Abelson virus macrophage tumour lines only showed a high degree of homology with the P388D1 line which is the prototype murine macrophage tumour line (Koren *et al.*, 1975). These cell lines also showed intermediate homology with the myelomonocytic WEH1-3B line. The latter also expresses the Mac-1 antigen (unpublished observation) and can be induced to differentiate into macrophages and granulocytes (Metcalf, 1979).

The only apparent anomaly encountered so far in the correlation between the glycoprotein pattern expressed by a cell line and the class of tumour from which it was derived was the P815 cell line. This has been classified as a mast cell neoplasm (Dunn & Potter, 1957) and yet it shows significant homology with the macrophage-type tumour lines. This might mean that the mast cell and macrophage neoplasms actually express similar patterns of glycoproteins or that the subline of the P815 line used was a contaminant macrophage-type cell. Studies are in progress to distinguish between these possibilities.

Apart from the above example, it appears that the pattern of Con A acceptor glycoproteins expressed by a tumour cell line is specific for the type of tumour from which it was derived.

### Discussion

These studies confirm that the Con A acceptor glycoproteins of cultured cells from both murine and human tumours are a relatively specific characteristic of each cell line. Broadly speaking, the glycoproteins on each fingerprint can be divided into two classes: constant glycoproteins which are expressed by all cell lines and variable glycoproteins which are not. Although not established formally, it appears that the constant glycoproteins from both human and murine sources are very similar. In contrast, the variable glycoproteins are an unusually specific characteristic of tumour cells and with the exception of cell lines derived from closely related tumours they are usually very different from different cell lines. Because the variable glycoproteins are usually the majority in a particular fingerprint the patterns from cell lines from unrelated tumours are easily distinguished from one another, rather as the peptide fingerprints from different proteins can be used to distinguish between proteins. Furthermore, just as peptide maps can be used to identify proteins the glycoprotein fingerprints can be used to identify various cultured cell lines. With the enormous proliferation of cultured tumour cell lines for laboratory investigation the problems of identification and cross-contamination have increased considerably (Gartler, 1968). Whilst enzyme (Povey *et al.*, 1976) and histocompatibility typing (O'Toole *et al.*, 1982) have proved very useful in cell identification they can be laborious and require specialised laboratory investigators. In contrast gel fingerprinting is virtually routine and can be adopted by almost any laboratory. One requirement of this approach to cell identification is that the phenotype should be stable during *in vitro* cell culture. Studies carried out so far show that this is indeed the case and in one detailed investigation of the stability of the glycoprotein pattern from a pancreatic adenocarcinoma (unpublished data) no significant instability was detected. Therefore the two-dimensional fingerprints of the Con A acceptor glycoproteins have the specificity and stability required to be useful in the identification of cultured tumour cell lines.

The most significant feature of the variable Con A acceptor glycoproteins of both murine and human tumour cell lines is the fact that they are not expressed in a completely idiosyncratic fashion. Thus, independently-isolated tumour cell lines can express very similar or even identical patterns of glycoproteins e.g. the MC6A and MC6B

**Figure 7** Cross comparison of the Con A acceptor glycoprotein fingerprints of 15 murine tumour cell lines. Comparison was carried out between pairs of maps prepared in parallel by direct superimposition and homology, estimated as described in **Materials and methods**. The cell lines used were: $T_1$ MBL2; $T_2$ EL4; T3 BW5147 (Glynn *et al.*, 1968); My X63 Ag8 (Horibata & Harris, 1970); Ab1 C57B110 5R; Ab2 C57B110 2R × 5R; Ab3 C57B110 A.A6; Ab4 C57B110BrA6; F1 C57B110MC6A; F2 C57B110MC6B (Sikora *et al.*, 1979); F3 SV40-Balb/c 3T3; F4 STO (Ware & Axelrad, 1972); Ms P815 (Dunn & Potter, 1957); $MF_1$ WEH1-3B (Warner *et al.*, 1969); $MF_2$ P388D1 (Koren *et al.*, 1975).

T = thymoma, My = myeloma, Ab = Abelson lymphoma, F = fibroblastoid, Ms = mastocytoma, MF = macrophage.

fibrosarcoma lines. This indicates that the set of variable glycoproteins in a particular pattern is expressed as a linked group of markers. Furthermore, these linked markers correlate with the type of tumour from which the cell lines are derived. Thus, the murine fibrosarcomas, murine macrophages, human EBV$^+$ lymphoblastoid lines and human carcinoma lines express patterns of glycoproteins which appear to be characteristic for cell lines derived from each class of tumour. Furthermore, in the murine fibrosarcomas the characteristic pattern of Con A acceptor glycoproteins is expressed by cell lines derived from tumours induced by different agents indicating that the mode of induction does not play a significant role in determining the pattern.

The three features of the Con A acceptor glycoproteins mentioned above, viz. their expression as a linked set, their correlation with the type of tumour involved and the apparent irrelevance of the tumour-inducing agent, all indicate that the patterns expressed by tumour cell lines are largely determined by the type of normal precursor cell from which each tumour is derived. This in turn suggests that the variable glycoproteins are indicators of the pathway of differentiation to which the pre-neoplastic precursor cell was committed. The interesting feature of the Con A acceptor glycoproteins as differentiation markers is the fact that each cell line expresses such a large number of such specific markers. Thus most cell lines. particularly the murine fibrosarcomas and macrophages express >50 separate Con A acceptor glycoproteins which are more or less characteristic for such cells. The biological function of these glycoproteins is not known. However, it has been speculated (Hood *et al.*, 1977) that cells might express surface markers at different stages of their normal lineages which could be important in their precise location in the organism and in the recognition of other cells and extracellular matrices. It is possible that the Con A acceptor glycoproteins serve such a role during normal development.

The linkage of the pattern of variable Con A acceptor glycoproteins to the state of differentiation of the normal pre-neoplastic precursor cell from which tumour cells are derived indicates that they may be particularly useful as specific markers for tumour cell classification. From this point of view they have some significant advantages. Thus, each class of tumour cell appears to express a large number of these markers all of which are revealed by a single analysis. Since the markers concerned are glycoproteins it is likely that many of them are expressed on cell surfaces, although the exact extent of this is not yet known. Consequently, specific antibodies may be used in simple binding assays to identify and classify the corresponding tumour cells. Of particular significance is the apparent specificity of the glycoprotein fingerprint for tumour cells derived from the same lineage but from precursor cells of differing maturity. For example the WEH1-3B cell, which is believed to originate from a relatively immature cell in the macrophage lineage, can be distinguished from the P388D1 cell which appears to be derived from a more mature cell. This may prove to be the most significant aspect of the Con A acceptor glycoproteins as tumour cell markers. Conventional classification of tumour cells is largely based on their expression of characteristics of mature cells from a particular lineage. However tumours probably arise from relatively immature cells (Potter, 1978; Mintz & Fleischmann, 1981) which may express mature markers to varying extents. In contrast, the variable glycoproteins do not appear to be expressed by mature cells such as lymphocytes and macrophages (manuscript in preparation) and may therefore be specific for the immature normal precursor cells. Thus, they could prove more specific as classification markers than the conventional markers being used currently.

Finally it is worth noting that although the Con A acceptor glycoproteins appear to be indicators of normal cell differentiation they may nevertheless prove useful as specific antigens for tumour diagnosis, localisation and therapy. This is based on the view that markers which have a relatively restricted expression amongst normal cells can be just as useful as classical tumour-specific markers because the clonal expansion which occurs during tumour growth will generate a corresponding expansion of the specific marker (Lampson & Levy, 1979). The use of Ig idiotypes in B lymphoblastoid tumours has often been quoted to exemplify this approach (Stevenson *et al.*, 1977). Thus, antibodies against the variable Con A glycoproteins could prove of considerable value in clinical investigations of tumours.

We thank all our colleagues, particularly A. Lowe and E. Lennox, for providing the many cell lines used in this study. J. Jarvis and C. Milstein provided the M1/70 antibody.

### References

ATKINSON, M.A.L. & BRAMWELL, M.E. (1981). Studies on the surface properties of hybrid cells III. A membrane glycoprotein found on the surface of a wide range of malignant cells. *J. Cell Sci.*, **48**, 147–170.

BASOMBRIO, M.A. (1970). Search for common malignant antigenicities among 25 sarcomas induced by methylcholanthrene. *Cancer Res.*, **30**, 2458–2464.

BRAMWELL, M.E. & HARRIS, H. (1978). An abnormal membrane glycoprotein associated with malignancy in a wide range of different tumours. *Proc. Roy. Soc. B.*, **201**, 87–106.

DER, C.J. & STANBRIDGE, E.J. (1981). A tumour-specific membrane phosphoprotein marker in human cell hybrids. *Cell*, **26**, 429–438.

DUNN, T.O. & POTTER, M. (1957). A transplantable mast cell neoplasm in the mouse. *J. Natl Canc. Inst.*, **18**, 587–601.

FOGH, J., FOGH, J.M. & ORFEO, T. (1977). One hundred and twenty-seven cultured human tumour cell lines producing tumours in nude mice. *J. Natl Canc. Inst.*, **59**, 221–226.

GARTLER, S.M. (1968). Apparent HeLa cell contamination of human heteroploid cell lines. *Nature*, **217**, 750–751.

GLYNN, J.P., MCCOY, J.L. & FEFER, A. (1968). Cross-resistance to the transplantation of syngeneic Friend, Moloney and Rauscher Virus-induced tumours. *Cancer Res.*, **28**, 438–439.

GRANT, A.G., DUKE, D. & HERMAN-TAYLOR, J. (1979). Establishment and characterisation of primary human pancreatic carcinoma in continuous cell culture and in nude mice. *Br. J. Cancer*, **39**, 143–149.

GREAVES, M.F. (1981). Analysis of the clinical and biological significance of lymphoid phenotypes in acute leukaemia. *Cancer Res.*, **41**, 4752–4766.

HOOD, L., HUANG, H.V. & DRYER, W.J. (1977). The area-code hypothesis: The immune system provides clues to understanding the genetic and molecular basis of cell recognition during development. *J. Supramol. Struct.*, **7**, 531–559.

HORIBATA, K. & HARRIS, A.W. (1970). Mouse myelomas and lymphomas in culture. *Exptl. Cell Res.*, **60**, 61–77.

KOCH, G.L.E. & SMITH, M.J. (1982). Analysis of the glycoproteins of murine tumour cell lines with $^{125}$I-Concanavalin A in two-dimensional electrophoresis gels. *Eur. J. Biochem.*, (in press).

KOREN, H.S., HANDWERGEN, B.S. & WUNDERLICH, J.R. (1975). Identification of macrophage-like characteristics in a cultured murine tumour line. *J. Immunol.*, **114**, 894–898.

LAMPSON, L.A. & LEVY, R. (1979). A role for clonal antigens in cancer diagnosis and therapy. *J. Natl Canc. Inst.*, **62**, 217–219.

METCALF, D. (1979). Clonal analysis of the action of GM-CSF on the proliferation and differentiation of myelomonocytic leukaemia cells. *Int. J. Cancer*, **24**, 616–623.

MINTZ, B. & FLEISCHMANN, R.A. (1981). Teratocarcinomas and other neoplasms as developmental defects in gene expression. *Adv. Canc. Res.*, **34**, 211–278.

O'FARRELL, P.H. (1975). High resolution two-dimensional electrophoresis of proteins. *J. Biol. Chem.*, **250**, 4007–4021.

O'TOOLE, C.M., TIPTAFT, R.C. & STEVENS, A. (1982). HLA antigen expression on urothelial cells: detection by antibody-dependent cell-mediated cytotoxicity. *Int. J. Cancer*, **29**, 391–395.

POTTER, V.R. (1978). Phenotypic diversity in experimental hepatomas: the concept of partially blocked ontogeny. *Br. J. Cancer*, **38**, 1–23.

POVEY, S., HOPKINSON, D.A., HARRIS, H. & FRANKS, L.M. (1976). Characteristics of tumour cell lines and differentiation from HeLa by enzyme typing. *Nature*, **264**, 60–63.

SIKORA, K., KOCH, G., BRENNER, S. & LENNOX, E. (1979). Partial purification of tumour-specific transplantation antigens from methylcholanthrene-induced murine sarcomas by immobilised lectins. *Br. J. Cancer*, **40**, 831–838.

SPRINGER, T., GALFRE, G., SECHER, D.S. & MILSTEIN, C. (1979). Mac-l: a macrophage differentiation antigen identified by monoclonal antibody. *Eur. J. Immunol.*, **9**, 301–307.

STEVENSON, G.T., ELLIOT, E.V. & STEVENSON, F.K. (1977). Idiotypic determinants on the surface immunoglobulins of neoplastic lymphocytes: a therapeutic target. *Fed. Proc.*, **36**, 2268–2271.

WARE, L.M. & AXELRAD, A.D. (1972). Inherited resistance to N- and B-tropic murine leukaemia viruses *in vitro*: Evidence that congenic mouse strains SIM and SIM.R differ at the FV-1 locus. *Virology*, **50**, 339–348.

WARNER, M.L., MOORE, M.A.S. & METCALF, D. (1969). A transplantable myelomonocytic leukaemia in Balb/c mice: cytology, karyotype and muramidase content. *J. Natl Canc. Inst.*, **43**, 963–982.